# Pressure-Driven Water Release from Magnesium Sulfate Hydrates: Thermodynamic and Mechanistic Insights

**DOI:** 10.1021/acs.inorgchem.5c03991

**Published:** 2025-10-28

**Authors:** Getachew G. Kebede, Ruth Franco, Fernando Izquierdo-Ruiz, Alvaro Lobato, J. Manuel Recio

**Affiliations:** † Center for Materials Science and Engineering, 37602 Addis Ababa University, Addis Ababa 3434, Ethiopia; ‡ Malta-Consolider Team and Department of Analytical and Physical Chemistry, 16763 University of Oviedo, Oviedo E-33006, Spain; § Malta-Consolider Team and Departamento de Química Física, 16734 Universidad Complutense de Madrid, Madrid E-28040, Spain

## Abstract

Understanding the behavior of hydrated salts under pressure is essential for interpreting geochemical processes in planetary interiors and for developing (de)­hydration-based technologies. In this study, we use density functional theory calculations to investigate the thermodynamics of pressure-induced dehydration in magnesium sulfate hydrates (MgSO_4_·*n*H_2_O, *n* = 11 and 7), where compression drives the release of water as dense ice polymorphs (such as ice II and VI) and the formation of hydrates with fewer water molecules. Our results show that dehydration becomes thermodynamically favorable at 0.8 GPa for MgSO_4_·11H_2_O and 1.1 GPa for MgSO_4_·7H_2_O, with ice VI emerging as the dominant crystallization product. Interaction energy analysis identifies interstitial, rather than metal-coordinated, water molecules as the dehydration initiation sites. Unlike thermal dehydration of MgSO_4_·7H_2_O, which proceeds via MgSO_4_·6H_2_O and water vapor, our calculations indicate that pressure-induced dehydration yields MgSO_4_·5H_2_O and dense ice. These results highlight distinct mechanisms of dehydration under temperature and pressure and provide insight into hydrate behavior relevant to both thermochemical technologies and planetary environments.

## Graphical abstract

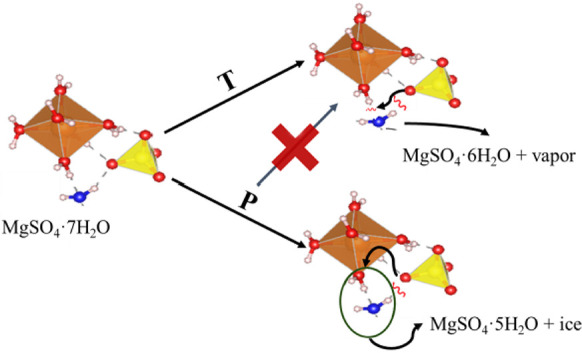

## Introduction

Hydrated salts, such as magnesium sulfate hydrates (MgSO_4_·*n*H_2_O) are of interest in various scientific and technological domains, ranging from planetary science to thermal energy storage. In planetary science, MgSO_4_ hydrates have been identified on the surfaces and subsurfaces of icy bodies such as Mars, Europa, and Ganymede, where they may form and transform under cryogenic and high-pressure conditions.^1–4^ On Earth, MgSO_4_·7H_2_O (epsomite) is considered a promising candidate for thermochemical energy storage.^5–11^ Their stability and transformation under varying pressure–temperature (*p*–*T*) conditions are essential in these areas.

A fundamental process affecting MgSO_4_·*n*H_2_O is pressure-induced dehydration, whereby water molecules are expelled from the crystal lattice, producing fewer hydrates and water. In high-pressure environments such as planetary interiors or cold, deep crustal settings, this released water can crystallize into ice, forming dense polymorphs like ice VI. In this context, dehydration is usually referred to as exsolution, with the released water remaining in liquid and/or solid form. These transformations are critical to understanding the deep water cycle in extraterrestrial contexts and are also of fundamental importance for their thermochemical energy applications.

The high-pressure behavior of MgSO_4_·7H_2_O (Epsomite), MgSO_4_·11H_2_O (Meridianiite), and MgSO_4_·1H_2_O (Kieserite) has received experimental attention.^12–20^ These studies reveal that MgSO_4_·7H_2_O and MgSO_4_·11H_2_O dehydrate under pressures below 2 GPa, with the liberated water typically crystallizing as ice VI, a dense high-pressure ice polymorph.^15–17^ For instance, MgSO_4_·11H_2_O has been observed to dehydrate into MgSO_4_·9H_2_O and ice VI near 0.9 GPa and 240 K.^16^ MgSO_4_·7H_2_O crystallizes in an orthorhombic structure (space group *P*2_1_2_1_2_1_), consisting of MgO_6_ octahedra and SO_4_ tetrahedra interconnected by hydrogen bonding.^14,21,22^ High-pressure studies reported a sequence of discontinuities upon compression that were interpreted as polymorphic phase transitions.^18^ However, other experimental work demonstrated that these discontinuities are associated with progressive dehydration (exsolution) reactions,^23^ in which epsomite transforms to MgSO_4_·5H_2_O and ice VI, and possibly through intermediates, yet unidentified, lower hydrates at 1.6 GPa and 293 K.^17^ Similar behavior is observed in other sulfate hydrates, such as CoSO_4_·7H_2_O and ZnSO_4_·7H_2_O, which dehydrate to lower hydrates and ice at 1–2 and 0.5 GPa, respectively.^24,25^ In the case of kieserite (MgSO_4_·1H_2_O), it transforms from a monoclinic (space group *C*2/*c*) to a triclinic (*P*1̅) phase at 2.72 GPa and 295 K^19^ without pressure-induced water release reported so far.

Computational studies are essential for gaining insights into these experimental observations and understanding the energetics and mechanisms governing pressure-induced dehydration processes. In this study, we address this concern by using a density functional theory (DFT) approach to model pressure-induced dehydration reactions in MgSO_4_·*n*H_2_O, focusing specifically on *n* = 7 and *n* = 11 hydration states, which have been the subject of specific high-pressure investigations within the MgSO_4_·*n*H_2_O systems.

Our aim is 2-fold. First, building upon our earlier work on the equations of state (EOS) of hydrated magnesium sulfates,^26^ we seek to determine the most thermodynamically favorable dehydration reactions. This task will be undertaken by evaluating enthalpy changes involving the formation of distinct ice polymorphs (Ih, II, VI, and VIII). Second, we analyzed local coordination environments and interaction energies of individual water molecules in MgSO_4_·*n*H_2_O (*n* = 7, 11) to identify structural patterns that explain the causes of dehydration. Our findings provide both thermodynamic and molecular-level insights into the pressure behavior of MgSO_4_ hydrates and offer valuable data and information on their transformation pathways under geologically relevant conditions, which are also useful for high-pressure materials design.

## Computational Details

In this work, we model coupled solid-state reactions involving MgSO_4_·*n*H_2_O dehydration under pressure with subsequent crystallization of water into ice polymorphs. Phase stability under hydrostatic pressure was evaluated using the static approximation (0 K calculations, neglecting zero-point energy). The thermodynamic reaction enthalpy was determined by enthalpy differences between reactants and products, Δ_*r*_ *H*(*p*). The enthalpy at pressure *p* is defined as *H*(*p*) = *E*(*V*) + *pV*. Entropy effects are expected to play a minor role in these solid–solid reactions involving crystals simulated with atoms at fixed positions. It is also worth noting that reactions yielding solid water are observed at temperatures below ambient. For these reasons, our Δ_*r*_ *H*(*p*) values might also be considered as good estimations of Δ_*r*_ *G*(*p*). The crystal structures of MgSO_4_·*n*H_2_O hydrates are available in the literature, and their corresponding *E* – *V* calculated data were obtained from our previous EOS study and used directly for the enthalpy evaluations presented here.^26^ Under compression (positive *p*), the volume of the system decreases, and its enthalpy increases (becomes less negative).

Given that ice VI has been experimentally observed as a dehydration product during pressure-induced dehydration of MgSO_4_·11H_2_O^15,16^ and MgSO_4_·7H_2_O,^17^ we model the structure and EOS of ice VI, but we also consider three other major ice phases (Ih, II, and VIII) to allow meaningful structural and energetic comparisons between low- to high-pressure regimes of ice stability at low temperature. In contrast to ices II and VIII, for which appropriate unit cell data for direct first-principles calculations, including hydrogen coordinates, are experimentally available, ice VI poses a challenge due to disordered hydrogen positions.

Ice VI crystallizes in a tetragonal structure (space group *P*4_2_/*nmc*) and contains 10 water molecules per unit cell.^27–30^ To calculate the *E* – *V* curve of ice VI, we first generated low-energy hydrogen-bonding configurations using a first-principles-based approach developed by Kuo and Kuhs^31^ and Komatsu et al.^30^ We performed a structural search with the unit cell parameters fixed to the experimentally determined values (*a* = *b* = 6.173 Å, *c* = 5.688 Å, α = β = γ = 90.0°^◦^, *V* = 216.764 Å^3^), and the oxygen atomic positions were constrained as obtained from Hirshfeld atom refinement of high-pressure single-crystal X-ray diffraction data by Chodkiewicz et al.^32^ Hydrogen atoms were initially placed along the lines connecting neighboring oxygen atoms, with O–H bond lengths set to 0.987 Å, ensuring compliance with the ice rules.^33,34^ Multiple hydrogen-bonding configurations satisfying the ice rules were generated for the unit cell. These configurations were then filtered using a graph algorithm^35^ to identify distinct arrangements.

DFT calculations were then performed to relax the atomic positions of these filtered configurations, while the unit cell dimensions were constrained to the experimental values. The lowest-energy configuration from the relaxed set was selected as the representative structure for the EOS calculations. While EOS parameters are typically derived by averaging across all low-energy configurations, we focused on the most stable structure in this study. This refined EOS was subsequently applied to model the pressure-induced dehydration of MgSO_4_·*n*H_2_O, focusing on the stability of ice VI as a dehydration product under high-pressure conditions.

For Ice Ih (hexagonal, *P*6_3_/*mmc* space group),^36^ a supercell containing 12 water molecules was constructed to account for hydrogen disorder. Since this Ih polymorph has been included in our study for the sake of completeness and does not play a significant role in the dehydration processes, we have not considered it necessary to repeat the same computational protocol as we did with Ice VI. For the other ice polymorphs, we adopted hydrogen-ordered structures available in the literature as input geometries: Ice II (rhombohedral, *R*3̅)^37^ and Ice VIII (tetragonal, *I*4_1_/*amd*).^27^ Nakamura and Ohtani^4^ demonstrated that Ice VII forms alongside Ice VI in the MgSO_4_–H_2_O system, and at low temperatures, the disordered Ice VII transforms into the ordered Ice VIII, which we used for our calculations.

All DFT calculations were performed using the VASP code^38–41^ employing the revised van der Waals density functional (rev-vdW-DF2),^42^ which has demonstrated good accuracy for hydrated materials, particularly in reproducing equilibrium hydrogen-bond distances and unit cell volumes.^26,43^ Projector augmented wave (PAW) potentials^41^ were used to describe core–valence interactions, with valence electron configurations of 2*p* ^6^3*s* ^2^ for Mg, 3*s* ^2^3*p* ^4^ for S, 2*s* ^2^2*p* ^4^ for O, and 1*s* ^1^ for H. A *k*-point sampling density corresponding to a spacing of 0.20 Å^–1^ was used for all calculations, along with a plane-wave energy cutoff of 600 eV.

To determine EOS parameters, we first calculated a set of (*E*_i_, *V*_i_) data points on a grid within a volume range considered by isotropically compressing (∼−20%) and expanding (∼+10%) the structures around their experimental equilibrium volumes. Then, the GIBBS code^44,45^ was used to fit the Vinet EOS^46^ to the calculated (*E*_i_, *V*_i_) data and to obtain the corresponding zero-pressure equilibrium volume (*V*_0_), bulk modulus (*B*_0_), and its pressure derivative $\left(B_{0}^{\prime }\right)$ (see ref 26 for more details). These parameters enabled the calculation of pressure–volume relations and the evaluation of the enthalpy of the hydrates involved in the dehydration reactions.

## Results and Discussion

### Ice Polymorphs: EOS and Stability

Our target is to investigate the pressure-induced dehydration reaction:

$${MgSO}_{4}\cdot nH_{2}O\left(s\right)\to {MgSO}_{4}\cdot mH_{2}O\left(s\right),+\left(n-m\right)H_{2}O\left(s\right)\left(n>m\right)$$ 1

One physically relevant question in this context is which ice polymorph forms upon dehydration under pressure. Since the water released from MgSO_4_·*n*H_2_O crystallizes into ice, the specific phase formed depends on *p* –*T* conditions. Experimental studies have reported that dehydration of MgSO_4_ hydrates occurs below 2 GPa, with the released water commonly assumed to crystallize as Ice VI.^16,17^ However, according to the known ice *p* –*T* phase diagram, this pressure range can stabilize multiple ice forms, including Ice Ih, II, III, V, VI, and IX, depending on the temperature.

To understand the behavior of water released during the pressure-induced dehydration of MgSO_4_·*n*H_2_O and to accurately evaluate the enthalpy changes associated with these reactions (as discussed in the following section Pressure-Induced Dehydration Reactions), it is essential to examine the EOS parameters of the reactants and products involved in eq 1 and, in particular, to compute how the enthalpy of ice polymorphs varies with pressure.

Table 1 presents the EOS parameters for four ice polymorphs (Ices Ih, II, VI, and VIII). The trend from Ice Ih to Ice VIII shows a systematic decrease in the molar volume (from 29.7 to 18.7 Å^3^) and an increase in the bulk modulus (from 16.0 to 23.5 GPa), reflecting progressive densification and stiffening of the hydrogen-bond network under pressure. We observed that the calculated phase transition pressures (last column in Table 1 and Figure 1) are systematically higher than the experimental values. For example, the Ih–II and II–VI transitions are calculated near 0.5 and 1.3 GPa, whereas experiments place them around 0.2 and 0.5 GPa.^30^ Ice VIII appears around 2.7 GPa in calculations compared to ∼2.0 GPa experimentally. These discrepancies are well-known and arise mainly from the use of DFT-GGA, as well as the omission of contributions from zero-point energy and finite temperature.^47,48^

**Table 1:** Calculated EOS Parameters of Selected Ice Polymorphs: Equilibrium Volume per Formula Unit (*V*_0_), Bulk Modulus (*B*_0_), and Its Pressure Derivative $\left(B_{0}^{\prime }\right)$ , All Evaluated at Zero Pressure^a^

Polymorph	*V*_0_ (Å^3^)	*B*_0_ (GPa)	$B_{0}^{\prime }$	Stability Range (GPa)
Ice Ih	29.689	16.016	5.591	0.000–0.558
Ice II	23.381	19.258	5.649	0.558–1.355
Ice VI	21.063	21.354	5.751	1.355–2.754
Ice VIII	18.740	23.503	6.291	2.754–3.250

a Also shown are the calculated pressure ranges that define the stability fields of the selected ice polymorphs. The upper limit for the Ice VIII polymorph (3.25 GPa) is the maximum pressure used in our simulations.

**Figure 1:**
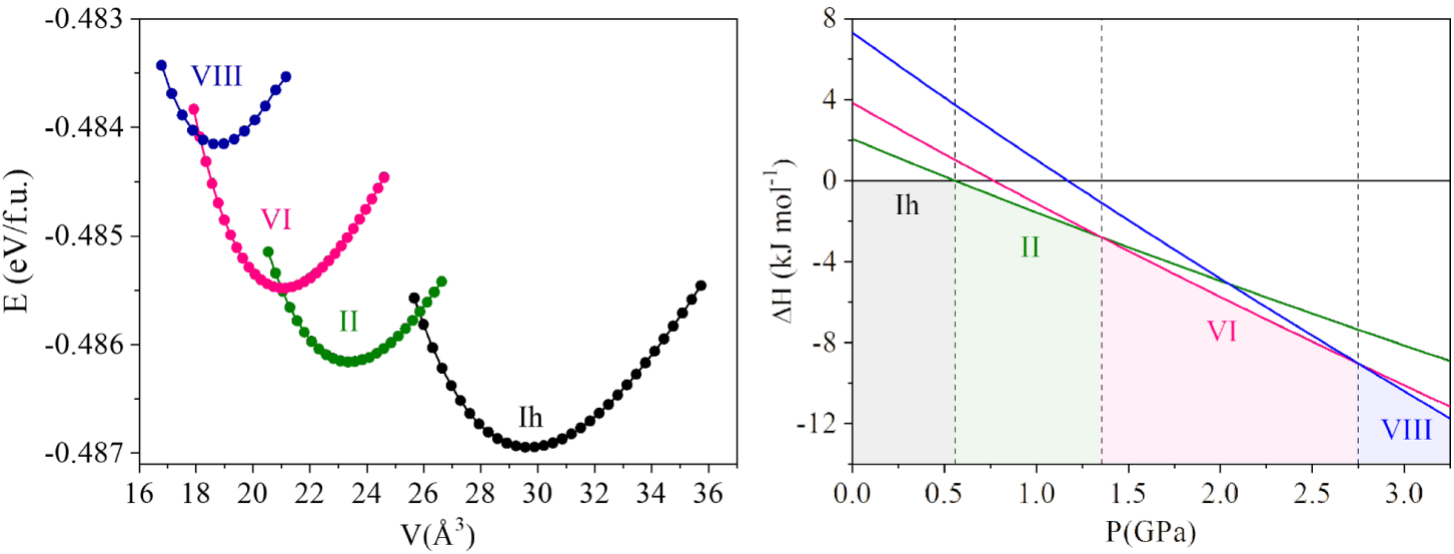
Calculated *E*(*V*) curves (left) and Δ*H* vs *p* (right) for the ice polymorphs considered in this study. In the right-hand panel, ice Ih is used as the reference phase, and vertical lines indicate the calculated phase boundaries, corresponding to the onset transition pressures of ice II, ice VI, and ice VIII.

Although our 0 K enthalpy calculations overestimate the transition pressures, they correctly reproduce the sequence of ice polymorph stabilities and are internally consistent for comparative purposes. Because full vibrational free energy calculations are computationally intensive, especially for large hydrate systems, we systematically apply a static enthalpy-based approximation to both ice and MgSO_4_·*n*H_2_O phases in this study.

### Pressure-Induced Dehydration Reactions

To model the experimentally observed pressure-induced dehydration reactions (as introduced earlier), we considered the following transformations:

2 $${\mathrm{MgSO}}_{4}\cdot 11H_{2}O\left(s\right)\to {\mathrm{MgSO}}_{4}\cdot 9H_{2}O\left(s\right)+2H_{2}O\left(s\right)$$

3 $${\mathrm{MgSO}}_{4}\cdot 7H_{2}O\left(s\right)\to {\mathrm{MgSO}}_{4}\cdot 5H_{2}O\left(s\right)+2H_{2}O\left(s\right)$$

The enthalpy change (Δ_*r*_ *H*) of each reaction was computed using the expression:

$$\begin{array}{c} \Delta_{r}H=\sum H_{\mathrm{products}}-\sum H_{\mathrm{reactants}} \end{array}=2H\left[H_{2}O\left(\mathrm{ice}\right)\right]+H\left[{\mathrm{MgSO}}_{4}\cdot mH_{2}O\right],-H\left[{\mathrm{MgSO}}_{4}\cdot nH_{2}O\right]$$ 4

where *n* = 11, 7 and *m* = 9, 5 for reactions ([Disp-formula eq2]) and ([Disp-formula eq3]), respectively. The enthalpy of water ice, *H*[H_2_O (ice)], was evaluated using the four polymorphs (Ice Ih, II, VI, and VIII). Two general approaches for calculating Δ_*r*_ *H* under pressure might be proposed: (i) the *fixed polymorph approach*, which uses a single ice phase (typically Ice VI, based on experimental precedent) across the entire pressure range or (ii) the *phase-field approach*, which selects the thermodynamically stable ice polymorph (Ih, II, VI, or VIII) appropriate for each pressure based on their pressure stability fields, as shown in Table 1. For greater thermodynamic rigor, we followed the latter approach. For the hydrate phases, ambient-pressure structures were used, except for *m* = 5, where a high-pressure structure from diffraction data was employed.

Figure 2 presents the calculated Δ_*r*_ *H* values for the reactions described in eqs 2 and 3 over the pressure range of 0–3.0 GPa. Table 2 summarizes the corresponding dehydration volumes (Δ*V*_deh_) and pressures (*P*_deh_) at which Δ_*r*_ *H* crosses zero, indicating the onset of a thermodynamic preference for dehydration. These values are reported for four crystalline ice polymorphs as dehydration products.

**Figure 2:**
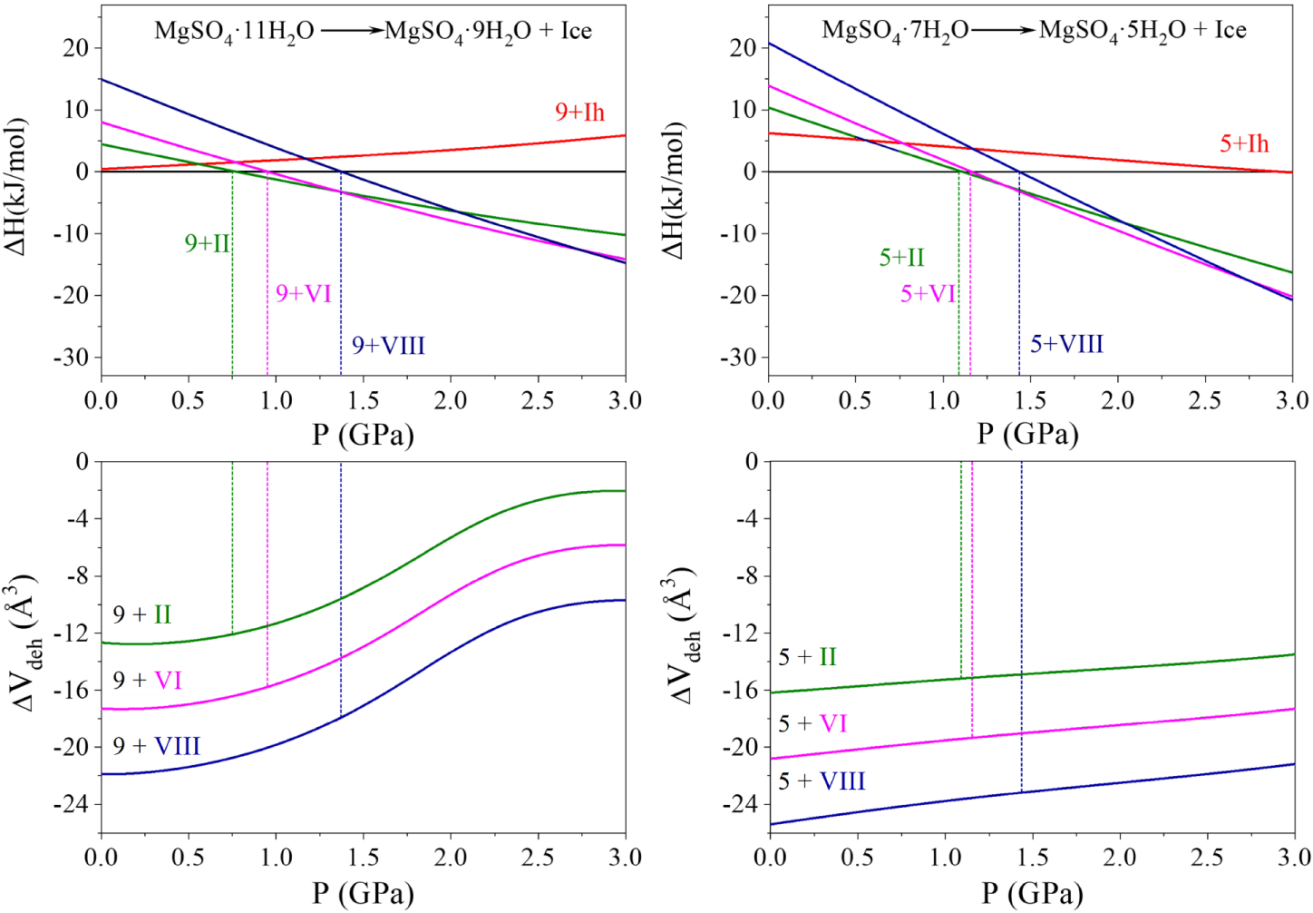
Calculated Δ_*r*_ *H* vs pressure (upper panels) for the dehydration reactions given by eq 2 (upper left) and eq 3 (upper right), and Δ*V*_deh_ vs pressure (lower panels) for eq 2 (lower left) and eq 3 (lower right). For the sake of clarity, hydrated states are labeled with the number of water molecules.

**Table 2:** Calculated Dehydration Pressures (*P*_deh_) and Corresponding Volume Changes (Δ*V*_deh_) at *P*_deh_ for the Dehydration of MgSO_4_·11H_2_O and MgSO_4_·7H_2_O^a^

Reaction	Δ*V*_deh_ (Å^3^)	*P*_deh_ (GPa)
MgSO_4_·11H_2_O → MgSO_4_·9H_2_O + 2H_2_O (ice Ih)	–	–
MgSO_4_·11H_2_O → MgSO_4_·9H_2_O + 2H_2_O (ice II)	–12.317	0.652
MgSO_4_·11H_2_O → MgSO_4_·9H_2_O + 2H_2_O (ice VI)	–16.190	0.834
MgSO_4_·11H_2_O → MgSO_4_·9H_2_O + 2H_2_O (ice VIII)	–18.730	1.233
MgSO_4_·11H_2_O → MgSO_4_·9H_2_O + 2H_2_O (ice, exp.^16^)	–	0.9
MgSO_4_·7H_2_O → MgSO_4_·5H_2_O + 2H_2_O (ice Ih)	–3.236	2.904
MgSO_4_·7H_2_O → MgSO_4_·5H_2_O + 2H_2_O (ice II)	–15.192	1.101
MgSO_4_·7H_2_O → MgSO_4_·5H_2_O + 2H_2_O (ice VI)	–19.362	1.156
MgSO_4_·7H_2_O → MgSO_4_·5H_2_O + 2H_2_O (ice VIII)	–23.207	1.432
MgSO_4_·7H_2_O → MgSO_4_·5H_2_O + 2H_2_O (ice, exp.^17^)	–	1.6

a Experimental values are included for comparison.

For MgSO_4_·11H_2_O (Figure 2, upper left panel), dehydration to MgSO_4_·9H_2_O accompanied by Ice II becomes favorable at 0.65 GPa. Dehydration leading to Ice VI and Ice VIII occurs at slightly higher pressures of 0.83 and 1.23 GPa, respectively. The calculated values for Ice VI agree well with the experimental *P*_deh_ of approximately 0.9 GPa,^16^ supporting the feasibility of pressure-induced dehydration under mild compression.

In the case of MgSO_4_·7H_2_O (upper right panel of Figure 2), a similar trend is observed, but with consistently higher *P*_deh_, indicating that greater compression is required to initiate dehydration compared to MgSO_4_·11H_2_O. As detailed in Section 3.3, this behavior is not unexpected as the ion–ion (Mg^2+^··· ${\mathrm{SO}}_{4}^{2-}$ ) and ion–water (Mg^2+^···OH_2_ and ${\mathrm{SO}}_{4}^{2-}$ ···H_2_O) interactions are stronger in MgSO_4_·7H_2_O than in MgSO_4_·11H_2_O. This is also reflected in the calculated values of *V*_0_ and *B*_0_, which are 238.83 Å^3^ and 28.17 GPa for MgSO_4_·7H_2_O, and 348.43 Å^3^ and 21.37 GPa for MgSO_4_·11H_2_O, respectively.^26^ As a result, the crystal structure of MgSO_4_·7H_2_O is more tightly bound, requiring higher pressure to overcome the energetic barriers for dehydration, and thus exhibits a higher dehydration pressure than MgSO_4_·11H_2_O.

The dehydration pressures for the formation of MgSO_4_·5H_2_O with Ice II, VI, and VIII are 1.10, 1.16, and 1.43 GPa, respectively. It is worth mentioning here that the experimental *P*_deh_ of 1.6 GPa (ref 17 is higher than the calculated values for Ice II–VIII transition, suggesting that additional factors such as structural constraints and kinetic effects can further complicate/delay the onset of the dehydration process.

The variation of the associated volume change in the dehydration reactions, defined as Δ*V*_deh_ = 2*V*_ice_ + *V*_*m*_ – *V*_*n*_ (where *m* = 9, 5 and *n* = 11, 7), with pressure is shown in the lower panels of Figure 2. The calculated Δ*V*_deh_ values corresponding to *P*_deh_ are presented in Table 2. Negative Δ*V*_deh_ values indicate that these dehydration reactions result in a net volume reduction, making the reaction thermodynamically favorable under pressure. This contraction becomes more pronounced when higher-density ice polymorphs form. For example, in the reaction MgSO_4_·7H_2_O → MgSO_4_·5H_2_O + 2H_2_O, the volume reduction is modest (−3.2 Å^3^) when Ice Ih forms, but increases to −23.2 Å^3^ when Ice VIII is the product. A similar trend is observed for the 11 → 9 dehydration reaction. These results illustrate that denser ice phases like Ice VI and Ice VIII contribute more to the overall volume reduction.

In this context, it is also instructive to consider the volume-based thermodynamic formalism developed by Glasser and Jenkins,^49–51^ who demonstrated a linear correlation between the number of water molecules (*n*) in a hydrate and the molar volume increase relative to the anhydrate. Our previous DFT results agree with a fitted slope of 25.3 Å^3^ per water molecule.^26^ Applying this model to the pressure-induced dehydration of MgSO_4_·*n*H_2_O, a linear volume reduction is expected as water is progressively removed (from 11 to 9, and 7 to 5), which adds a systematic thermodynamic driving force for dehydration under pressure, independent of the ice phase formed. However, the reduction in hydrate volume alone is not sufficient to drive the reaction. For example, if Ice Ih is the dehydration product in the 11 → 9 reaction, the reaction remains thermodynamically unfavorable within the pressure range investigated. Notice that the zero-pressure volume of a water molecule in the Ice Ih phase (29.689 Å^3^, see Table 1) is greater than the value in the hydrated salts. For the other water ice polymorphs, this is not the case.

This interplay between the intrinsic hydrate volume behavior and the density of the resulting ice polymorphs directly impacts the thermodynamic preference for dehydration. From the thermodynamic relation *H* = *E* + *p*Δ*V*, the volume collapse contributes a negative *p*Δ*V* term to the enthalpy change (Δ_*r*_ *H*), effectively lowering the reaction enthalpy as pressure increases. Thus, pressure favors dehydration reactions by reducing the volume.

These findings highlight the role of the ice phase in determining the pressure at which dehydration occurs. At ambient conditions, dehydration is typically endothermic: significant energy is required to overcome water–ion interactions (Mg^2+^···OH_2_ and ${\mathrm{SO}}_{4}^{2-}$ ···H_2_O), water–water hydrogen bonds, and to convert bound water into vapor. Under pressure, two main factors reverse this energetic behavior: (i) the structural reorganization into more compact, lower-volume hydrate phases (MgSO_4_·*n*H_2_O → MgSO_4_·*m*H_2_O, *n* > *m*), which is thermodynamically favored via the −pΔV term and (ii) the formation of dense, energetically stable solid ice polymorphs instead of high-enthalpy vapor. In other words, the linear volume reduction tied to the lowering of the hydration level and the stabilization of denser ice polymorphs are the main elements that drive pressure-induced dehydration reactions.

### Signatures of Early-Stage Pressure-Induced Dehydration

To gain insight into the initial stages of pressure-induced dehydration reactions, we provide in Figure 3 a detailed visualization of the coordination environments of crystallographically distinct water molecules in MgSO_4_·7H_2_O and MgSO_4_·11H_2_O, extracted from DFT-optimized geometries based on experimental diffraction data.^14,18,21,22,52,53^ This figure helps to analyze the interaction energies of individual water molecules and H-bonded dimers in the hydrated salts.

**Figure 3:**
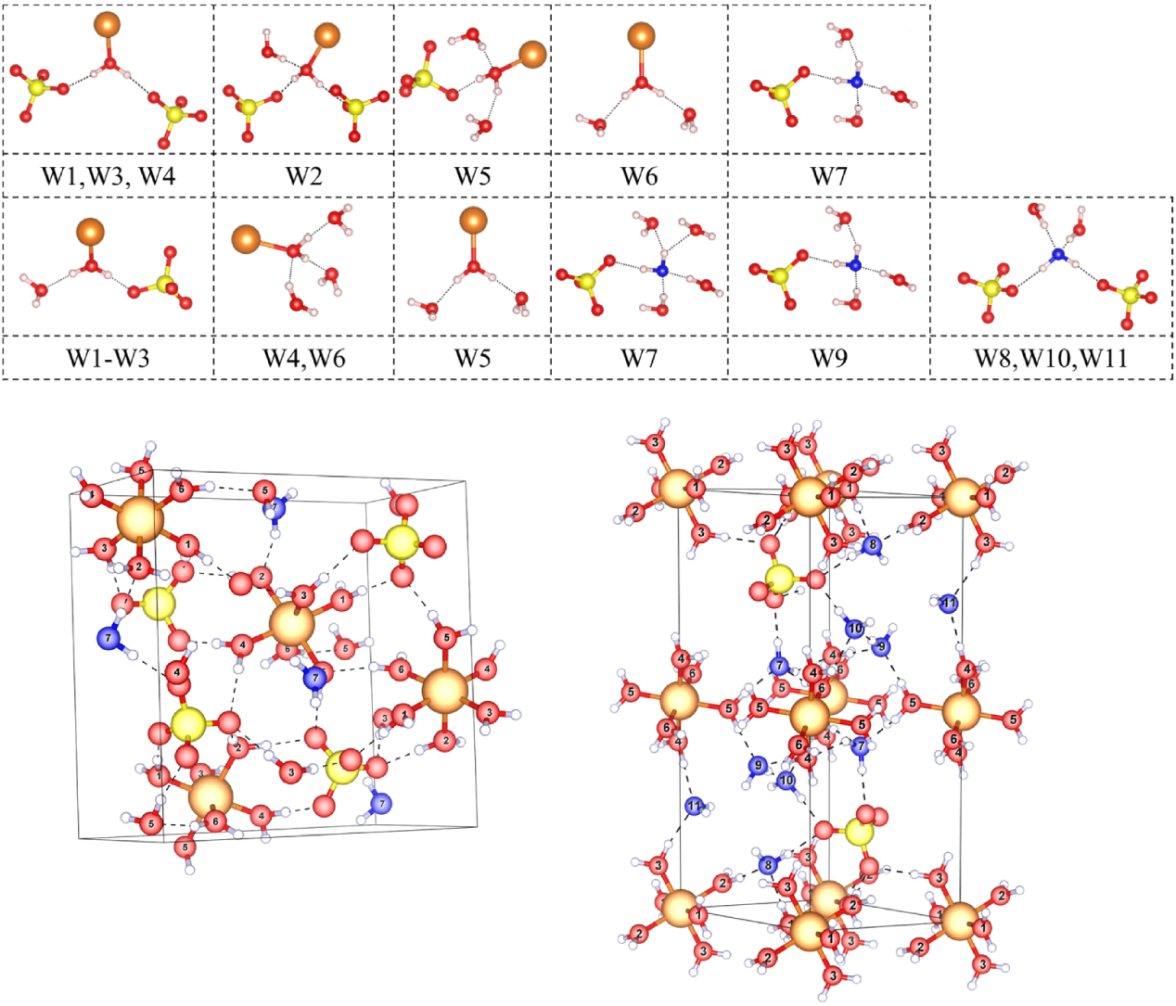
Water coordination environments in bulk MgSO_4_·7H_2_O and MgSO_4_·11H_2_O. The upper panel shows the coordination environments for all crystallographically unique water molecules, labeled W1 to W7 for MgSO_4_·7H_2_O (first row) and W1 to W11 for MgSO_4_·11H_2_O (second row), following the labeling convention of the experimental authors. The bottom panels display the bulk structures of MgSO_4_·7H_2_O (left) and MgSO_4_·11H_2_O (right). Atoms: Mg = orange, S = yellow, H = gray, and O = red when coordinated to sulfur and for noninterstitial water (W1–W6 in both MgSO_4_·7H_2_O and MgSO_4_·11H_2_O) while interstitial water oxygen atoms are denoted in blue (W7 in MgSO_4_·7H_2_O and W7–W11 in MgSO_4_·11H_2_O). Numbers on oxygen atoms correspond to the water molecules specified in the upper panel.

In the upper panel of Figure 3, the first row illustrates the coordination motifs for W1–W7 in MgSO_4_·7H_2_O. Water molecules W1, W3, and W4 share similar geometries, each donating hydrogen bonds to neighboring sulfate groups. All six (W1–W6) are directly coordinated to Mg^2+^, completing the formation of MgO_6_ octahedra. In contrast, W7 (shown in blue) is an interstitial water molecule that is not bonded directly to Mg^2+^. It serves as both a hydrogen bond donor and acceptor, reinforcing the local hydrogen-bonding network and contributing to lattice cohesion without participating in metal coordination.

The second row of Figure 3 displays the coordination motifs for W1–W11 in MgSO_4_·11H_2_O. W1–W6 closely resemble those in the heptahydrate, participating in octahedral coordination and acting as hydrogen bond donors to adjacent sulfate groups or water molecules. W7–W11 (shown in blue) are interstitial and do not form direct bonds with Mg^2+^. Instead, they establish an extended hydrogen-bonding network, bridging coordination units and enhancing three-dimensional connectivity. These interstitial water molecules typically accept hydrogen bonds from two other water molecules via their oxygen atoms, and most also donate hydrogen bonds to SO_4_ tetrahedra.

The bottom panel of the figure compares the bulk structures of MgSO_4_·7H_2_O (left) and MgSO_4_·11H_2_O (right). The structure of MgSO_4_·7H_2_O is relatively compact, with most water molecules directly coordinating with Mg^2+^ and a limited interstitial content. Hydrogen bonding primarily links sulfate tetrahedra and water molecules into extended chains. On the other hand, MgSO_4_·11H_2_O features a more open and flexible framework due to the presence of additional interstitial water molecules.

The interaction energies (*E*_int_) of water molecules in MgSO_4_·*n*H_2_O provide insight into the initial stages of dehydration under pressure. We define *E*_int_ as:

5 $$E_{\mathrm{int}}=E_{\mathrm{tot}}\left[{\mathrm{MgSO}}_{4}\cdot nH_{2}O\right]-E_{\mathrm{tot}}\left[{\mathrm{MgSO}}_{4}\cdot \left(n-1\right)H_{2}O\right]-E_{\mathrm{tot}}\left[H_{2}O\right]$$

where *E*_tot_[MgSO_4_·*n*H_2_O] is the total energy of the optimized hydrated structure, and the remaining terms represent the single-point energies of the corresponding crystal with one water molecule removed and of an isolated water molecule. This quantity represents the energetic cost of removing a specific water molecule from the crystal without relaxation of the surrounding lattice. Thus, lower *E*_int_ values indicate more weakly bound water molecules and highlight sites likely involved in the onset of dehydration under pressure. The calculated *E*_int_ values for the individual water molecules and the possible H-bonds in the two hydrates are given in Table 3 at 0 GPa and at pressures slightly above the corresponding *P*_deh_ into ice VI.

**Table 3:** Calculated Interaction Energies (in kJ/mol Water) for Individual Water Molecules and Hydrogen Bonds in MgSO_4_·11H_2_O and MgSO_4_·7H_2_O at Two Different Pressures: Ambient and Near Their Respective Dehydration Pressures^a^

MgSO_4_·11H_2_O					
Water	0 GPa	0.9 GPa	H-bond	0 GPa	0.9 GPa
W1	151.84	148.13	W1···W8	123.37	121.69
W2	154.78	149.09	W2···W8	129.21	125.70
W3	146.16	142.92	W3···W11	112.90	109.85
W4	187.63	186.93	W4···W11	154.78	153.50
W5	165.15	163.13	W4···W9	132.81	131.47
W6	166.24	161.93	W5···W7	122.28	119.30
W7	134.62	129.18	W5···W9	135.99	133.89
W8	138.07	135.78	W6···W7	131.09	127.28
W9	148.73	143.50	W6···W10	139.56	135.86
W10	150.60	145.18	W7···W4	150.84	147.43
W11	124.34	120.77	W7···W6	143.22	138.46
			W9···W10	137.02	133.563

MgSO_4_·7H_2_O					
Water	0 GPa	1.4 GPa	H-bond	0 GPa	1.4 GPa
W1	169.26	162.67	W5···W7	138.196	135.39
W2	168.23	159.14	W6···W5	150.651	148.51
W3	160.76	150.03	W6···W7	134.524	133.09
W4	164.97	152.30	W7···W2	128.202	121.48
W5	184.23	181.17			
W6	170.82	169.58			
W7	144.86	141.05			

a The calculated *E*_int_ values at each pressure correspond to structures whose pressures were obtained by fitting the energy-volume data.

In MgSO_4_·11H_2_O, *E*_int_ values at 0 GPa range from approximately 124.3 kJ/mol (W11) to 187.6 kJ/mol (W4), with a clear distinction between coordinated and interstitial waters. With the exception of W10, the interstitial water molecules consistently exhibit the lowest *E*_int_ values. Upon compression to 0.9 GPa, these values decrease slightly (e.g., W11 from 124 to 121 kJ/mol), maintaining the same energetic ranking. On average, the removal of hydrogen-bonded dimers tends to result in lower *E*_int_ values compared to the removal of individual water molecules, suggesting that cooperative cluster removal may be thermodynamically more favorable than isolated water detachment.

MgSO_4_·7H_2_O shows higher interaction energies across all water molecules compared to MgSO_4_·11H_2_O. All water molecules except W7 participate in Mg coordination, forming part of the rigid MgO_6_ octahedral framework. W7 is the sole interstitial water, participating in three hydrogen-bonded dimers (W5···W7, W6···W7, and W7···W2), each with relatively low *E*_int_ values ranging from 124 to 138 kJ/mol. Upon increasing pressure to 1.4 GPa, these dimers show a further reduction in interaction energy, particularly W7···W2 and W5···W7. In contrast, the coordinated waters (W1–W6) remain strongly bound (*E*_int_ > 150 kJ/mol).

These results highlight that the presence and distribution of interstitial waters are essential for initiating pressure-induced dehydration. MgSO_4_·11H_2_O contains five interstitial waters and exhibits a lower dehydration onset pressure, while MgSO_4_·7H_2_O, with only one interstitial water, requires higher pressures for dehydration. Structural analysis (Figure 3) supports this interpretation: interstitial waters engage in extended hydrogen-bonded networks without directly coordinating to Mg^2+^, resulting in weaker binding and a greater preference for pressure-induced release.

This observation raises an important question: if dehydration initiates with the detachment of interstitial water, why does MgSO_4_·7H_2_O dehydrates directly to MgSO_4_·5H_2_O^17^ bypassing the hexahydrate *C*2/*c* phase.^54,55^ Our calculations provide mechanistic insight. Hydrogen-bonded water dimers exhibit lower interaction energies than isolated molecules, suggesting that water release proceeds cooperatively via cluster detachment ((H_2_O)_*2n*_ → ice) rather than stepwise release (2*n*(H_2_O)_1_ → ice) under pressure. In MgSO_4_·7H_2_O, the W7-centered hydrogen-bond network, specifically the dimers W5···W7, W6···W7, and W7···W2, would be the initiation site for this process according to our calculations. Note that the interaction energies of water dimers in Table 3 are reported on a per-mole-of-H_2_O basis to allow direct comparison with monomer interaction energies.

Detachment of such dimers requires breaking one of the coordinated bonds (from W5, W6, or W2), reducing the Mg coordination number from six to five. This creates both coordination and structural voids within the lattice. However, these disruptions can be stabilized through the coordination of nearby ${\mathrm{SO}}_{4}^{2-}$ oxygen atoms to Mg^2+^ effectively restoring the MgO_6_ octahedron. The energy required to break the original Mg-water bond is partially or possibly fully offset/compensated by the energy gained from forming new Mg^2+^– ${\mathrm{SO}}_{4}^{2-}$ interactions. We believe this is thermodynamically favorable, as the oxygen atoms of ${\mathrm{SO}}_{4}^{2-}$ are more negatively charged than those of the coordinated water oxygen.

This leads to a follow-up question: which structural form of the pentahydrate is actually formed during this pressure-induced dehydration? MgSO_4_·5H_2_O exists in two polymorphs: an ambient-pressure *P*1 phase^56^ and a high-pressure orthorhombic *Pna*2_1_ dehydration product of MgSO_4_·7H_2_O, identified by Wang et al.^17^ In the latter, all five water molecules are coordinated to Mg^2+^, while the *P*1 phase contains one interstitial water. Our analysis supports the observation of the orthorhombic phase. If a single coordinated water molecule is removed via hydrogen bonding with an interstitial partner, then it could be replaced by a SO_4_ oxygen to maintain the MgO_6_ octahedral integrity, yielding a fully coordinated five-water structure, as observed in the *Pna*2_1_ phase. Conversely, the formation of the ambient-pressure *P*1 structure from MgSO_4_·7H_2_O would require the loss of two coordinated water molecules, as it retains one interstitial water. However, our *E*_int_ analysis indicates that H-bonded water molecules (W6···W5) require higher energy for detachment, rendering this pathway less favorable (see Table 3).

The dehydration of MgSO_4_·7H_2_O can also be triggered by temperature. This fact raises a third indirect but related question. How do these two, in principle, opposing effects on material structure (temperature and pressure) influence the dehydration pathways? Generally, temperature promotes lattice expansion, whereas pressure induces structural densification. However, in the case of MgSO_4_·7H_2_O, both thermal and compression strains lead to dehydration, albeit with different dehydration products. This contrast demonstrates the fundamentally distinct mechanisms that come into play.

Under thermal conditions, MgSO_4_·7H_2_O undergoes a well-characterized, multistep dehydration process: it first transforms into the hexahydrate, then proceeds through amorphous intermediates before eventually reaching the anhydrous phase.^5–11^ The initial step, occurring between 25 and 55 ^◦^°C^5^ involves the release of the most weakly bound water molecule, i.e, the one identified as W7, resulting in the formation of MgSO_4_·6H_2_O. This process is endothermic under ambient pressure and requires sufficient thermal energy to break Mg^2+^–H_2_O coordination bonds, as well as H_2_O···SO_4_ and H_2_O···H_2_O hydrogen bonds and van der Waals interactions. Moreover, additional energy is needed to overcome the enthalpy of vaporization, as the detached water exits the crystal as a vapor. As a result, thermal dehydration proceeds stepwise, preferentially removing the most weakly bound water molecules and forming MgSO_4_·6H_2_O as the first intermediate.

Under pressure, MgSO_4_·7H_2_O directly forms the MgSO_4_·5H_2_O *Pna*2_1_ phase. As detailed in section Pressure-Induced Dehydration Reactions, dehydration under pressure becomes thermodynamically favorable due to the negative *p*Δ*V* contribution to the enthalpy and the formation of dense ice phases (Ice VI or Ice VIII) instead of water vapor. H-bonded water dimers involving W7 and one coordinated water can be expelled collectively from the lattice and stabilized as ice. For completeness, we also calculated the dehydration of MgSO_4_·7H_2_O to MgSO_4_·6H_2_O under pressure at 0, 1.5, and 2 GPa. The calculated Δ_*r*_ *H* values are +9.35, +7.05, and +4.59 kJ/mol, respectively, indicating that this reaction remains thermodynamically unfavorable, though the enthalpy becomes less positive with increasing pressure. Nevertheless, experimental observations suggest that an unidentified intermediate phase may exist, which could involve a high-pressure polymorph of MgSO_4_·6H_2_O in equilibrium with liquid water (or aqueous solution).^23^ Such a phase would alter the *p*Δ*V* contribution, deserving further thermodynamic modeling in future work. Since our study only evaluated enthalpies with respect to crystalline ices, we cannot rule out such a pathway.

It is also important to recognize that pressure- and temperature-driven dehydration mechanisms occur under fundamentally different conditions. Pressure-induced dehydration takes place under geological stresses relevant to planetary interiors,^16,17^ while temperature-driven processes are pertinent to thermochemical technologies.^5^ Together, these insights bridge the understanding of MgSO_4_ hydrate behavior in both industrial and planetary science contexts. While *E*_int_ offers thermodynamic insight, it neglects lattice relaxation, finite temperature, and dynamic effects. We believe that future studies need to combine DFT with molecular dynamics or kinetic Monte Carlo simulations to elucidate the mechanism and kinetics of these pressure-induced dehydration reactions.

## Conclusions

In this work, we have performed enthalpy calculations using density functional theory under static conditions to investigate the thermodynamics and microscopic mechanisms of pressure-induced dehydration in magnesium sulfate hydrates (MgSO_4_·*n*H_2_O, *n* = 7 and 11). Four ice polymorphs, namely Ice Ih, II, VI, and VIII, were considered as possible water crystallization products.

Our main findings are as follows:

- Dehydration becomes thermodynamically favorable at moderate pressures, occurring at 0.65 GPa for MgSO_4_·11H_2_O and 1.1 GPa for MgSO_4_·7H_2_O when denser ice phases such as Ice II or VI are formed. The preference of dehydration is governed by two main factors: (i) volume reduction due to lattice densification of the hydrate, contributing with a stabilizing −*pΔV* term to the enthalpy decrease and (ii) formation of dense, low-enthalpy ice polymorphs, instead of water vapor as in thermal dehydration.
- Interaction energy analysis shows that interstitial water molecules and hydrogen-bonded dimers are the most weakly bound components, identifying them as likely initiation sites for dehydration. MgSO_4_·11H_2_O, which contains five interstitial water molecules, dehydrates more readily than MgSO_4_·7H_2_O, which has only one.
- Dehydration under pressure proceeds cooperatively via the detachment of weakly bound water dimers, rather than through stepwise release of individual molecules. This process is accompanied by local structural rearrangements that preserve Mg^2+^ coordination through substitution by SO_4_ oxygens.
- Pressure-induced and thermal dehydration follow distinct mechanistic pathways. Under pressure, MgSO_4_·7H_2_O dehydrates directly to MgSO_4_·5H_2_O and crystalline ice, bypassing the hexahydrate observed in thermal dehydration. This contrast reflects the fundamentally different thermodynamic landscapes governed by pressure versus temperature.

All in all, these results provide predictive insights into the stability and transformation behavior of hydrated magnesium sulfates under high-pressure conditions relevant to planetary interiors. They also offer design principles for pressure-tuned solid-state reactions in functional hydrate materials.
